# Reducing False-Positives in Lung Nodules Detection Using Balanced Datasets

**DOI:** 10.3389/fpubh.2021.671070

**Published:** 2021-05-19

**Authors:** Jinglun Liang, Guoliang Ye, Jianwen Guo, Qifan Huang, Shaohui Zhang

**Affiliations:** ^1^School of Mechanical Engineering, Dongguan University of Technology, Dongguan, China; ^2^School of Electromechanical Engineering, Guangdong University of Technology, Guangzhou, China

**Keywords:** pulmonary nodule detection, lung image classification, deep learning, convolutional neural network, transfer learning

## Abstract

Malignant pulmonary nodules are one of the main manifestations of lung cancer in early CT image screening. Since lung cancer may have no early obvious symptoms, it is important to develop a computer-aided detection (CAD) system to assist doctors to detect the malignant pulmonary nodules in the early stage of lung cancer CT diagnosis. Due to the recent successful applications of deep learning in image processing, more and more researchers have been trying to apply it to the diagnosis of pulmonary nodules. However, due to the ratio of nodules and non-nodules samples used in the training and testing datasets usually being different from the practical ratio of lung cancer, the CAD classification systems may easily produce higher false-positives while using this imbalanced dataset. This work introduces a filtering step to remove the irrelevant images from the dataset, and the results show that the false-positives can be reduced and the accuracy can be above 98%. There are two steps in nodule detection. Firstly, the images with pulmonary nodules are screened from the whole lung CT images of the patients. Secondly, the exact locations of pulmonary nodules will be detected using Faster R-CNN. Final results show that this method can effectively detect the pulmonary nodules in the CT images and hence potentially assist doctors in the early diagnosis of lung cancer.

## Introduction

Lung cancer is one of the most common cancers in the world and has the highest proportion of new cases (11.6%) and deaths (18.4%) among all cancers in 2018 (1–3). A lack of obvious clinical symptoms in the early stage of the disease are one of the main reasons for the high mortality rate of lung cancer, because most patients have missed the best time for treatment when they go to see a doctor with symptoms. At that time, lung cancer may be too advanced to be effectively treated with surgery. Therefore, the early screening, diagnosis, and treatment of lung cancer is a major focus of lung cancer prevention and control. In the early stage, malignant pulmonary nodules are one of the main manifestations of lung cancer on CT images. Pulmonary nodules are a common disease, which is a small round or oval tissue growing in the lungs. However, it does not mean all pulmonary nodules are malignant, so it is necessary to detect the malignant pulmonary nodules in the lung.

Previous studies show that low-dose spiral CT (CT) is an effective method to analyze lung cancer nodules and reduce mortality compared with chest X-ray photography (4). The whole lung usually needs to be scanned to detect tiny pulmonary nodules, which will produce a huge amount of images for each patient. It is a serious challenge for radiologists to detect pulmonary nodules from so many CT images by excluding uncorrelated tissues such as bronchi and blood vessels. It could easily lead to misdiagnosis due to the radiologist's fatigue and distraction caused by the overwhelming number of images. Therefore, it is urgent to develop an automated efficient system for pulmonary nodules screening.

A Computer-Aided-Detection (CAD) system is one of the feasible methods to assist doctors to detect and classify lung images. Furthermore, a promising CAD system not only can reduce the missed detection of pulmonary nodules but also can improve the accuracy and efficiency of the analysis of CT images. There are several commercial CAD systems in clinical use, such as the Lung Care commercial CAD system developed by Siemens and the Image Checker CTLN-1000 commercial CAD system developed by R2 (5). A typical CAD system mainly consists of 3 parts, including pre-processing, nodule candidate detection, and false-positive reduction (6). The main purpose of pre-processing is to standardize data, enhance images and reduce noise. The detection stage after pre-processing uses very high sensitivity to detect all nodules as much as possible, forming candidate nodules with many false positives. The previous stage produces a large number of candidate nodules, so this stage is mainly to reduce the number of false positives in the candidate and generate the final result.

In the past decade, especially in combination with image processing methods (7), machine learning methods have been studied extensively in the medical detection field. In addition, researchers have also obtained many related achievements (8, 9). The pulmonary nodules detection in lung CT images is one of the complex and highly concerning problems in medical image processing. The conventional lung nodule detection algorithms detect the pulmonary nodules by processing and analyzing CT images mainly through combining the pulmonary nodule characteristics such as size, location, and shape with the image processing algorithm and machine learning (10). These detection algorithms include two steps. The region of interest (ROI) was firstly extracted by image segments to identify suspected targets, and secondly, the ROI will be classified as malignant and non-malignant pulmonary nodules. Messay et al. pre-processed the image by combining the multi-layer gray threshold method with the open operation in morphology and then extracted the ROI of pulmonary nodules by rule-based analysis method (11). Murphy et al. used cluster structure and cluster merging to extract the nodules by calculating the shape index and curviness of each pixel in the image of the lung parenchyma, and setting thresholds for these two parameters to obtain seed points (10). Although conventional machine learning methods can achieve high accuracy in one type of target, they are still hard to achieve good results in other types of modules. This is mainly due to the complexity of different types of pulmonary nodules and the complex situations in classification.

With the development of artificial intelligence (AI) and the increasingly reliable large-scale annotated datasets, a series of deep learning algorithms has experienced an enormous advance in the image processing and video processing field, especially the deep convolutional neural network (CNN). Meanwhile, many improvements and optimization algorithms were proposed to improve the effect (12), and it has gradually made achievements in the field of medical image recognition (13, 14). CNN can automatically learn image features from sufficient training data. Automatic extraction of pulmonary nodules features can adapt to different types of pulmonary nodules. It can avoid the problems of omission or incomplete features in artificial extraction features and also improve the automation of pulmonary nodules detection (15). Therefore, compared with the traditional computer-aided lung image diagnosis method the deep learning algorithm has great advantages. It shows a significant improvement in the detection of the pulmonary nodules and it is gradually becoming the mainstream method in the field of pulmonary nodules detection and more and more research results have been achieved.

Anirudhi et al. proposed a method based on a 3D CNN for pulmonary nodules detection. It used weakly labeled data to train the neural networks in the field of pulmonary nodules detection. The experimental results were also superior to the traditional methods and the method produced fewer false positives (16, 17). Golan et al. proposed a method for lung nodule detection based on a deep CNN, which used the open data set LIDC and IDRI with a CNN to extract the characteristics of the lung nodules. This method does not use the segmentation and false-positive method, but still obtains a good result (15). Fu et al. proposed a feature extraction method of pulmonary nodules based on CNN. This method can effectively extract the brightness, shape, material, and other features of pulmonary nodules. This method can effectively combine the features extracted by hand with the features extracted by the neural network (18). Li et al. used an integrated CNN to solve the problem of high false-positives. This method combined three CNNs and achieved good results on the JSRT data set (19). Although deep learning can detect pulmonary nodules with various characteristics, it still produces many false positives. Thus, the high false-positive rate is the key issue of using deep learning techniques in pulmonary nodules.

This paper intends to solve the mentioned problem from the perspective of preliminary screening. The main idea is to introduce a filtering step to remove irrelevant images before testing to reduce false positives. This new method is based on deep learning which is divided into two steps. The first step is to screen the images with pulmonary nodules from the whole lung CT image of the patients and the second step is to detect the exact locations of pulmonary nodules using Faster R-CNN. In the first step, a classification network is trained to screen the target images with suspected pulmonary. The experimental results on a balanced LUNA16 dataset have achieved an accuracy of above 98%. In this way, the suspected pulmonary nodules in the CT image of the case can be quickly selected by the classification network, and the location range of the suspected nodules in the CT image can be detected by the FasterRCNN. This can improve the efficiency of the detector, increase the reliability of the detection, and reduce the false positive of the detector.

The main framework of this paper is as follows. In section Background, we briefly introduce the relative networks, including Alexnet, Resnet, and FasterRCNN. In section Materials and Methods, we describe the LUNA16 dataset, the CT image processing, and the experimental method. In section Experimental Results and Analysis we explain the experimental results and analysis. The last section is the conclusion of this paper.

## Background

### Transfer Learning

Because of the increasing popularity of deep neural networks, more and more scholars are adopting deep learning to solve complex problems in the field of medical imaging. At present, there are mainly three feasible methods to successfully apply CNNs to medical images: (1) Training CNN from the ground up, (2) conducting unsupervised CNN pre-training with the supervised fine-tuning base on off-the-shelf pre-trained CNN features, (3) transfer learning (20). Since the pre-trained network has learned a wealth of image features, it is usually faster and easier to use the pre-trained network with transfer learning compared to training the network from scratch. ImageNet is a famous database in the ImageNet Large-Scale Visual Recognition Challenge (ILSVRC), and the majority of the pre-trained networks are trained on it (21). It can learn features specific to the new data set when fine-tuning the network. In the following, we introduce the networks that were used in this paper, Alexnet Faster R-CNN and ResNet.

### Alexnet

For screening suspected pulmonary, we train a classification model base on Alexnet in the first step. Alexnet is a typical deep convolutional neural network and the champion of the ImageNet 2012 Image Recognition Challenge. The Alexnet network has a total of 60 million parameters and 650,000 neurons, consisting of five convolutional layers and three fully-connected layers. After the first, third, and fifth convolutional layers are followed by the max-pooling layer, the last fully-connected layer has a 1000-way softmax (22). The main feature of the Alexnet networks includes the following four parts:

1. The Relu activation function is added at the end of each Conv layer, which solves the problem of gradient disappearance of Sigmoid and makes the convergence faster. The common linear rectifier functions are the ramp function, which is
  (1) $$\begin{array}{l} f\left(x\right)=\max\left(0,x\right) \end{array}$$
2. Here x is the input to the neuron.
3. To reduce overfitting, it employed the random discard technique (Dropout) in the fully-connected layers. Dropout is regularization method in which some neural network units are temporarily discarded (their weights are retained) from the network according to a certain probability and no longer respond to the forward and reverse transmission of data in the training process of the deep learning network. At the same time, the data set is artificially enlarged, including image translation, horizontal reflection, and changing the intensity of the RGB channel in the training image.
4. It has also added a layer of Normalization (Local Response Normalization), which makes it more accurate. ReLU function does not have a limited range like tanh and Sigmoid, so it needs to be normalized after ReLU. The idea of LRN originated from a concept called lateral inhibition in neurobiology, which means that the activated neuron inhibits the surrounding neurons.
5. The core idea of Local Response Normalization is to normalize by using neighboring data, as shown in the following formula:
  (2) $$\begin{array}{c} b_{x,y}^{i}=a_{x,y}^{i}/(k+\alpha \sum_{j=\max(0,i-n/2)}^{\min(N-1,i+n/2)}{\left(a_{x,y}^{i}\right)}^{2}{)}^{\beta } \end{array}$$
  Where $a_{x,y}^{i}$ acts on the convolution kernel at position (*x, y*), and then performs ReLU, the resulting neuron output. *N* is the total number of convolution kernels of this layer. *n* is the number of convolution kernels adjacent to the same location. *k*, α, β are hyper-parameters.
6. It used the overlapping maximum pool and obtain greater performance. In general, there will not be overlap between adjacent sliding windows, which means the pooling unit's step size s equal to the pooling unit's size z. On the contrary, it set s < z in Alexnet networks.

### Faster-RCNN

In fact, detecting pulmonary nodules is one of the target detection of lung images. The target detection methods based on deep learning are mainly divided into two categories: (1) end-to-end, such as YOLO and SSD network architecture, (2) based on Region Proposal like Faster R-CNN. The former has a relatively fast detection speed, while the latter has a relatively high detection rate and accuracy. Therefore, the detection network of this paper adopted Faster R-CNN as the basic network structure.

The feature of the target detection method based on Regional Proposal is to extract the regional proposal from the input image at first, which is to get the Region Of Interest (ROI) of the target. Before the Faster R-CNN was proposed, the common method for obtaining Regional Proposal is Selective Search. Target detection methods such as R-CNN, SPP-NET, Fast R-CNN, etc., use selective search algorithms to extract target regions. Compared with the former algorithm, the groundbreaking development of Faster R-CNN directly computes the candidate box by Region Proposal networks, which makes the target detection speed significantly improved.

The network structure of Faster R-CNN mainly includes four parts:

1. Conv layers. Faster R-CNN as a detection method based on the CNN network target, this part is the basis of the extraction of the features of images, consisting of Conv + ReLU + pooling layer. These feature maps obtained from Conv layers are shared for subsequent Region Proposal Networks (RPN) layer and full connection layer.
2. Region Proposal Networks (RPN). The Region Proposal is generated by the RPN network. This part uses Softmax to classify anchors as positive or negative proposals and then obtains accurate proposals through bounding box regression correction.
3. Anchors. The key in RPN is anchors, which is a set of rectangular boxes, in which each row has four values (x1, y1, x2, y2), representing the coordinates of the upper left corner and the lower right corner of the rectangle. Input candidate boxes of different sizes can be obtained by setting different aspect ratios of the anchor. These anchors actually introduce the multi-scale approach that is often used in detecting.
4. ROI Pooling. This part combines the feature maps and Region Proposals from the output of Conv layers and RPN layer, integrating the information to extract proposal feature maps and send them to the subsequent full connection layer to determine the target category. The exact position of the anchor is obtained again by the bounding Box regression.

The Loss used by the entire network is as follows:

(3) $$\begin{array}{l} L\left(\left\{p_{i}\right\},\left\{t_{i}\right\}\right)=\frac{1}{N_{cls}}\underset{i}{\sum }L_{cls}\left(p_{i},p_{i}^{*}\right)+\lambda \frac{1}{N_{reg}}\underset{i}{\sum }p_{i}^{*}L_{reg}\left(t_{i},t_{i}^{*}\right) \end{array}$$

In the above formula, *i* represents the anchor index in the mini-batch and *p*_*i*_ is positive softmax probability. Moreover, $p_{i}^{*}$ represents the corresponding ground-truth predict probability, that is, when IOU >0.7 is between the anchor and ground-truth, the anchor is considered to be positive and $p_{i}^{*}=1$; on the contrary, when IOU <0.3, this anchor is considered as negative and $p_{i}^{*}=0$; anchors with 0.3< IOU <0.7 are excluded. *t*_*i*_ represents the predicted bounding box and $t_{i}^{*}$ represents the ground-truth box corresponding to the positive anchor. The total loss is divided into two parts. For the regression loss, it is activated only for the positive anchor. The two terms using *N*_*cls*_ and *N*_*reg*_ to normalize, since in the actual process, the gap between *N*_*cls*_ and *N*_*reg*_ is too large, use the parameter λ to balance (23).

### ResNet

He Kaiming's team proposed a deep convolutional neural network structure ResNet in 2015. Furthermore, the network won the champion of image classification, object detection, and target positioning in the ImageNet (ILSVRC2015) competition.

As CNN can extract the features of multiple levels from the data, and that the more network layers mean can extract richer features at different levels. Therefore, the deeper the network structure, the stronger the ability to extract abstract features, and the richer the semantic information obtained. However, when the depth of the network is increased, the effect of the stochastic gradient descent algorithm will become weaker, which will eventually lead to a gradient disappearance or gradient explosion. The previous network structure can train dozens of layers of the network by standard initialization and regularization layer, but with the further increase of the network layer, the degradation phenomenon will eventually appear, namely as the increase of the number of the network layer the accuracy of both the training set and the test set decreases. It is not caused by overfitting, but by redundant network layers learning parameters that are not identical mappings.

The idea of ResNet is to assume that there is an optimized network layer in a network layer, so often the deep network we design has many redundant network layers. So we want these redundant layers to be able to do identity mapping so that the inputs and outputs that go through the identity layer are exactly the same (24).

The ResNet still allows the non-linear layer to satisfy *H*(*x*, ω_*h*_), and then introduces a short connection directly from the input to the output of the non-linear layer, making the whole mapping to

(4) $$\begin{array}{l} y=H\left(x,\omega_{h}\right)+x \end{array}$$

This is the core formula of the ResNet. In other words, the ResNet is an operation of network construction, and any network that uses this operation can be called the residual network. Through the experiment on ImageNet, the ResNet can be deepened to hundreds of layers, and higher accuracy of previous convolutional neural networks such as VGGNet and GoogLeNet can be obtained.

## Materials and Methods

### Image Datasets

In this study, we make use of the LUNA16 (LUng Nodule Analysis) datasets for training and testing the deep learning model, which is one of the most representative international data sets in the field of pulmonary nodule detection, providing a publicly available data set about pulmonary nodules in the lung. In LUNA16, the data was collected from LIIDC-IDRI data sets, which is the largest public database for lung nodules (25–27). Based on LIDC-IDRI, LUNA16 datasets retain scans with a thickness of ≤ 3 mm and eliminate inconsistent or missing slices. Thus, 888 CT scans were screened out of 1,018 scans in LIDC-IDRI. In addition, four experienced thoracic radiologists annotated all LIDC-IDRI scans and there were 36,378 annotations made by the radiologists in these 888 scans. However, only the annotations were categorized as pulmonary nodules larger than 3 mm as relevant lesions. So only the pulmonary nodules that had been annotated by at least three out of the four radiologists were selected.

Finally, 888 scans were screened in the LUNA16 data set. A total of 1,186 nodules were annotated by at least three radiologists, which are lesions that the algorithm should detect. For ease of download, the data is not stored in the generic DOM format, but in Metalmage (.mhd) format, with each image the size of 512×512 pixels.

### Image Pre-processing

According to the principle of CT, the value of CT represents that X-ray beams illuminate different parts of the body with different densities to distinguish different tissues and organs. The higher the value of CT, the greater the density of the substance, and HU (Hounsfield unit) is the unit of CT value. The lung CT value is between −600 and −450 HU, the body fat is between −20 and −10 HU, blood is between 13 and 32 HU. For reference, in the state of nature, the CT value of the air is about −1,000 HU, and water is about 0 HU, the bone of the human body is about 1,000 HU. The raw CT images include other substances like air and skeleton that we do not focus on, so we filter out the irrelevant substance by a threshold of the CT value to obtain clear images.

**Figure 1:**
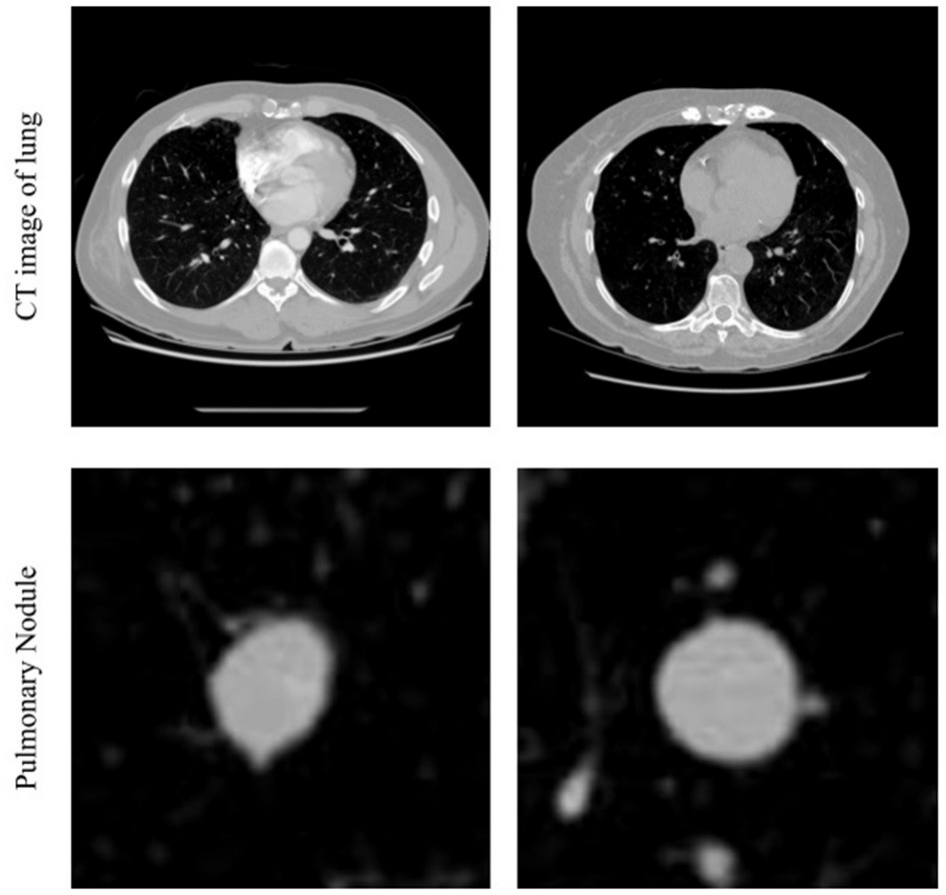
Example of CT images in lung and pulmonary nodule.

We read the CT image from each subset of Luna16, then filtered the values of < −1,000 HU and >500 HU, and standardized to the range of 0–1. After that, we were able to get the CT images with clear pulmonary nodules, which are shown in Figure 1 first row. The center of the image in the second row in Figure 1 shows the pulmonary nodules.

### Training

To accurately screen out images with pulmonary nodules, images with pulmonary nodules were taken as positive samples, whereas images without pulmonary nodules were taken as negative samples. To test the effect of sample balance on the model, several models were trained according to different positive and negative sample ratios. In this study, all experiments are conducted on a computer with an Intel Xeon Silver 4210 CPU and two NVIDIA GeForce RTX 2080 Ti GPU, and the software environment is Win10 and MATLAB 2020a.

The details of the experiment setting are as follows:

1. In the training dataset, images from LUNA16 datasets were mainly divided into the following two parts: The positive samples taken from the 1,186 CT images with annotations by the radiologists mentioned earlier. The negative samples mainly consist of total lung CT images from several cases that were not annotated. According to the different proportions of the number of positive samples and negative samples, several different groups were constructed for training and testing their respective effects.
2. To adapt the pre-training network to the new data set, the network was fine-tuned as follows: The last three layers of the network were removed and replaced with a fully connected layer, a softmax layer, and a classification output layer. The output of the fully connected layer was set to 2, which is the same size as the number of classes in the new data, and both WeightLearnRateFactor and BiasLearnRateFactor were 20.
3. All the samples used for training were resized to 227^*^227 pixels to adapt the input layer of the pre-train network Alexnet. The data set is divided into the training set and test set for cross-validation. The mini-batch size was set to 128, the learning rate was 0.001, and the max epoch number was 200.

After filtering the images, we trained the Faster R-CNN model to detect pulmonary nodules. According to the annotation information of 1,186 pulmonary nodules, the pulmonary nodules were annotated to generate the annotation files needed for training. After the selective node ROI was extracted from the RPN, Resnet101 was used to determine whether the region was a nodule or not. The training option was mainly as follows, the optimization method was stochastic gradient descent method, 500 epoch, 1 mini-batch size, 0.0001 initial learning rate, 0.1 “LearnRateDropFactor,” 100 “LearnRateDropPeriod.”

## Experimental Results and Analysis

### Results on Difference Proportion Sample

We conducted computer experiments on the LUNA16 dataset to prove the effectiveness of the method. Table 1 summarizes the performance of the model with different ratios of training data, including accuracy, True Positive Rate (TPR), and False Positive Rate (FPR). It is obvious that the accuracy could reach a maximum of 99.43% in the first row of Table 1 when the ratio of Pulmonary nodule and Non-Pulmonary nodule is near 1, that is, the number of positive samples and negative samples is balanced (28).

**Table 1:** Summary of inference different ratio of training data, presenting the accuracy.

**Number of images**		**Evaluate**		
**Positive**	**Negative**	**Accuracy**	**TPR**	**FPR**
1,086	1,171 (4 cases)	99.43%	98.90% (347 TP/351 P)	0% (0 FP/351 N)
1,086	1,353 (5 cases)	99.08%	98.00% (344 TP/351 P)	0% (0 FP/406 N)
1,086	1,029 (4 cases)	99.39%	98.90% (347 TP/351 P)	0% (0 FP/309 N)
1,086	10,166 (50 cases)	97.68%	78.06% (274 TP/351 P)	0% (0 FP/3050 N)
1,086	67,845 (287 cases)	98.91%	32.48% (114 TP/351 P)	0.01% (3 FP/20350 N)
201	201(1 case)	99.20%	98.30% (59 TP/60 P)	0% (0 FP/60 N)
494	494(2 cases)	98.00%	95.90% (142 TP/148 P)	0% (0 FP/148 N)
753	753(3 cases)	99.30%	98.70% (223 TP/226 P)	0% (0 FP/226 N)

*TP, True Positive; P, Positive; FP False Positive; N, Negatives*.

In addition to this, it does not perform very well in the case of unbalanced proportions of samples in the training set. Due to the serious imbalance of samples, networks are prone to treat the test sets as a category with a large sample size. Although the accuracy performance is high, the recall rate is low. As can be seen from Table 1, when the ratio increases from 1:1 to 1:10 and then to 1:60, the TPR also decreases continuously. Because of the unbalanced sample proportion, increasing the sample size will lead to a worse effect. However, there were only 1,086 positive samples, so the generalization ability of the model is not strong.

### Results on Detection

Then we introduced the CT images of the patients into the classification network and classified several CT images before and after the location of pulmonary nodules. After determining the range of pulmonary nodules, the Faster RCNN model was used to detect pulmonary nodules.

**Figure 2:**
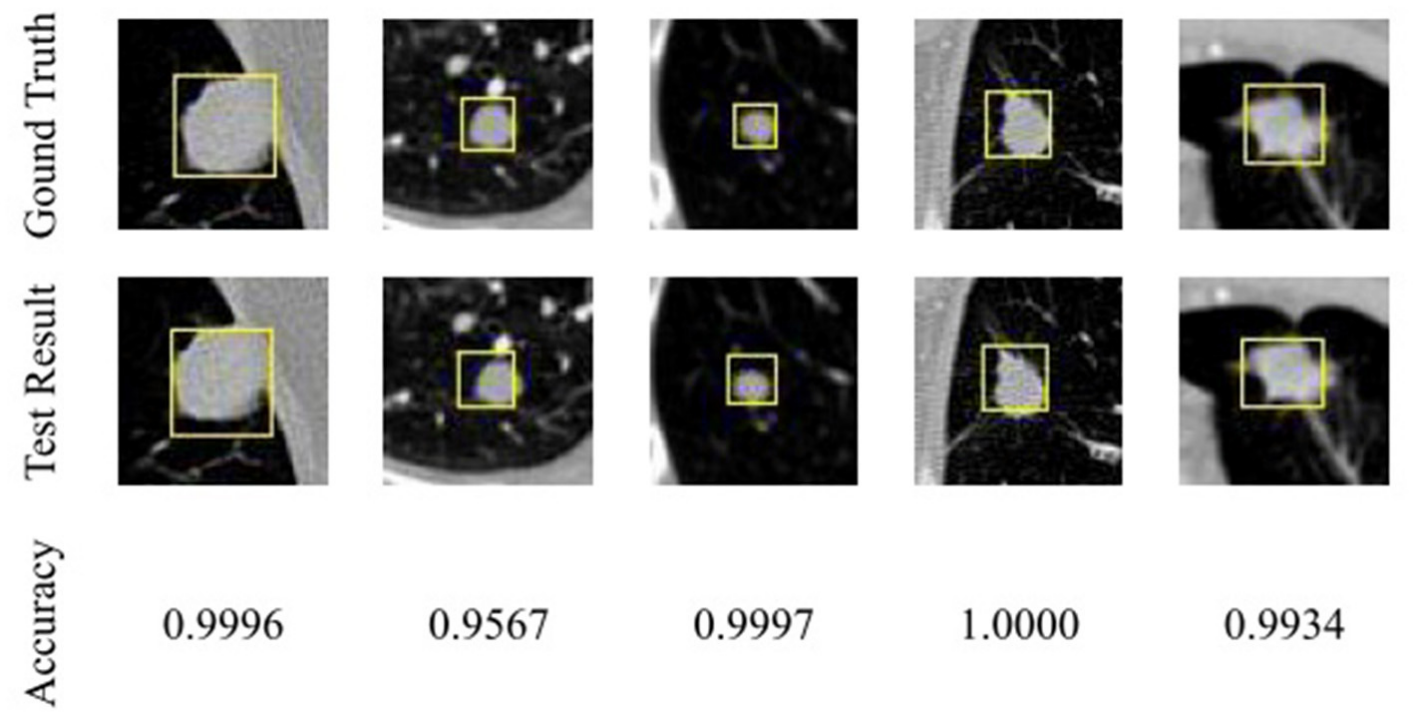
Comparison of pulmonary nodules test results and actual locations.

We visualize the results of the test on the test set to verify their accuracy. The test results were shown in Figure 2, in which CT images with pulmonary nodules were selected from the test set for testing. The first row in the figure is marked with the annotation by radiologists, the second row is detection network test results, and the third row is the nodules detection accuracy.

As you can see from Figure 2, the detected nodule is approximately the same as the actual location, and the size of the detection frame is approximately the same as the actual size. Detection accuracy has reached a high value, which can prove that based on the detection of the network of the nodules, the test set has a good detection effect.

### Discussion

According to the result of the tests on the LUNA16 datasets, the pulmonary nodule classification model performance is satisfactory and stable when the proportions of samples are relatively balanced. It means that CT images with positive pulmonary nodules can be efficiently and accurately classified through the classification network. Therefore, a simple filtering process can remove a lot of unrelated images so that the Faster R-CNN model could detect the pulmonary nodule in a small quantity of CT images that may contain pulmonary nodules without examining all CT images. So, it is a feasible method and is expected to help solve the problems of low detection rates and high false-positives in pulmonary nodules detection.

In this way, the suspected pulmonary nodules in the CT image of the case would quickly be selected through the classification network, and the location range of the suspected nodules in the CT image can be determined, and then detected by the detector, so as to improve the efficiency of the detector, increase the reliability of the detection, and reduce the false positive of the detector. It means that it could effectively detect the positive pulmonary nodules in the total CT image to assist doctors in the early screening of pulmonary nodules and has certain research value.

## Conclusions

In this paper, deep learning and related algorithms are used to study and discuss lung image classification, a new method based on the deep convolutional neural network to detect pulmonary nodules in CT images is proposed. This method can be regarded as a different way to reduce the false-positive rate of detection by Screening targets before detection. The main contribution of this method greatly reduces the detection time and efficiency and reduces the false-positive rate. The following conclusions can be drawn from the experimental results:

1. Under the training set of positive and negative sample balance, the classification network can accurately classify lung CT images with pulmonary nodules or not.
2. A simple filtering process can remove a large number of unrelated images so that the Faster R-CNN model could detect the pulmonary nodule in a small quantity of CT images without examining all CT images. Therefore, the proposed method can reduce the time spent on testing and improve efficiency.
3. Because the number of samples of pulmonary nodules is insufficient, the generalization ability of the model is not strong.

In the next work, we intend to improve the generalization ability. Ideally speaking, if we can introduce more positive samples to the training dataset, the predictive performance can be more accurate. Moreover, we hope to integrate the information about benign or malignant pulmonary nodules into the deep network so that it can automatically complete testing and evaluation.

## Data Availability Statement

Publicly available datasets were analyzed in this study. This data can be found here: https://luna16.grand-challenge.org.

## Ethics Statement

Ethical review and approval was not required for the study on human participants in accordance with the local legislation and institutional requirements. Written informed consent for participation was not required for this study in accordance with the national legislation and the institutional requirements.

## Author Contributions

JL proposed the research question, conducted the analysis, and edited the manuscript. GY and JG did the investigation, validated the results, discussed, and gave advice. QH accomplished the dataset preparation, model training, and wrote the original draft. SZ contributed to the associated analysis and design of experiments, reviewed, and edited the manuscript. All authors contributed to the article and determined the submitted version together.

## Conflict of Interest

The authors declare that the research was conducted in the absence of any commercial or financial relationships that could be construed as a potential conflict of interest.
